# Prognostic value of serum uric acid and tumor response to induction chemotherapy in locally advanced nasopharyngeal carcinoma

**DOI:** 10.1186/s12885-021-08285-7

**Published:** 2021-05-08

**Authors:** Yuanji Xu, Zijie Wu, Wangzhong Ye, Youping Xiao, Wei Zheng, Qinyan Chen, Penggang Bai, Zhizhong Lin, Chuanben Chen

**Affiliations:** 1 Department of Radiation Oncology, Fujian Medical University Cancer Hospital, Fujian Cancer Hospital, No.420, Fuma Road, Fuzhou, Fujian 350014 People’s Republic of China; 2 Department of Radiology, Fujian Medical University Cancer Hospital, Fujian Cancer Hospital, Fuzhou, Fujian People’s Republic of China

**Keywords:** Nasopharyngeal carcinoma, Serum uric acid, Tumor response, Prognosis

## Abstract

**Background:**

To explore the combined predictive value of serum uric acid (SUA) and tumor response to induction chemotherapy (IC) in locally advanced nasopharyngeal carcinoma (LANPC) patients receiving IC followed by concurrent chemoradiation therapy (CCRT).

**Methods:**

A total of 341 LANPC patients treated with IC + CCRT were enrolled in this retrospective study. Overall survival (OS), progression-free survival (PFS), locoregional relapse-free survival (LRFS), and distant metastasis-free survival (DMFS) were compared by the Kaplan-Meier analysis and the log-rank test, and multivariable survival analysis was carried out to investigate the independent prognostic factors.

**Results:**

Univariate analysis showed that a low SUA level and unsatisfactory tumor response to two cycles of IC both were negative predictors for OS, PFS, and DMFS in patients with LANPC. multivariable analysis demonstrated that the SUA level after two cycles of IC was an independent prognostic factor for OS (*P* = 0.012) but of borderline significance for PFS and DMFS (*P =* 0.055 and *P =* 0.067, respectively). Furthermore, tumor response to IC was of independent significance for predicting OS, PFS, and DMFS, respectively. Finally, LANPC patients with satisfactory tumor response and a high SUA level after two cycles of IC had a better OS, PFS, and DMFS than those with unsatisfactory tumor response and a low SUA level.

**Conclusion:**

The SUA level and the tumor response to two cycles of IC had predictive value for LANPC patients treated with IC plus CCRT. However, more aggressive therapeutic strategies are recommended for those with a low SUA level and unsatisfactory tumor response to two cycles of IC.

## Background

Nasopharyngeal carcinoma (NPC) is a distinct malignant tumor with the highest number of incidences reported in South China. It is diverse from other types of head and neck squamous cell carcinoma in regard to epidemiology, biological characteristics, and clinical treatment [1]. The concurrent chemoradiotherapy (CCRT) followed by adjuvant chemotherapy or induction chemotherapy (IC) in addition to CCRT for locally advanced nasopharyngeal carcinoma (LANPC) was proposed as level 2A evidence by the National Comprehensive Cancer Network (NCCN) guidelines [2]. The increasing number of randomized controlled trials has demonstrated that the addition of IC to CCRT is of great importance in the treatment of LANPC patients in the intensity-modulated radiation therapy (IMRT) era, which reduces distant metastasis and subsequently improves the overall survival (OS) [3–5]. Nevertheless, 20% ~ 30% patients with NPC would develop locoregional or distant metastasis, and distant metastasis was the most commonly seen failure pattern after treatment [6]. Therefore, explore novel prognostic factors to guide the clinical decision-making for a favorable and precise treatment after IC is an urgent requirement.

To date, the investigation on the prognostic factors of LANPC patients receiving IC remains largely unknown. In 2015, it was reported that measurable EBV-DNA loads as well as unfavorable tumor response (stable disease or disease progression) to IC were validated as negative predictors for LANPC patients [7]. In 2016, the tumor response to IC was subsequently determined as an independent prognostic factor for LANPC patients with IMRT [8]. In 2018, neutropenia during the first cycle of IC was found to be predictive for the poor survival of LANPC patients [9]. Recently, plasma EBV DNA load at IC completion was verified to be a robust and earlier survival outcome predictor for LANPC patients [10]. These observations confirmed that both plasma EBV-DNA load and the tumor response to IC had predictive value in LANPC patients that may be applied to direct the risk stratification and early treatment modification. Hence, in addition to plasma EBV DNA load, it is necessary to explore other biochemical indicators and predict the survival of patients with LANPC along with tumor response.

Uric acid is the final product of purine metabolism, which acts as a main antioxidant in serum and plays a vital role in defending cells from free radical-induced damage [11]. A previous study suggested that elevated uric acid levels might be attributed to increased purine metabolism by the action of xanthine oxidase, produced as a result of RNA-DNA breakdown in patients receiving radiotherapy [12]. In addition, the consumption of early appearing neutrophils in trauma or tumor lysis syndrome caused by radiochemotherapy could also elevate the serum uric acid (SUA) levels [13]. Moreover, aberrant SUA levels were involved with survival outcome in NPC patients in the IMRT era. Reportedly, the post-treatment SUA level was highly predictive of outcome in NPC patients treated by IMRT [14]. The pretreatment SUA level was an important biomarker in predicting distant metastasis in LANPC patients receiving IMRT [15]. Nevertheless, the predictive value of the SUA level after IC or the SUA levels associated with the tumor response to IC in LANPC patients undergoing IMRT has not yet been determined.

The present study aims to confirm whether the level of SUA after IC has prognostic significance in patients with LANPC. Furthermore, the level of SUA combined with tumor response to IC was investigated to evaluate the combined predictive value and guide the risk stratification for the decision of proper therapeutic schemes in LANPC.

## Materials and methods

### Patients and pretreatment evaluation

All 341 pathologically diagnosed NPC patients free of distant metastasis were enrolled in this retrospective study that was approved by Fujian Medical University Cancer Hospital institutional review board with a waiver of informed consent. All patients were treated with IC plus CCRT at our hospital from September 2014 to May 2017. The entry criteria were as follows: 1) records of the SUA levels of post-induction chemotherapy; 2) underwent a second MRI after two cycles of IC; 3) complete clinical data; 4) eliminate hyperuricemia or gout before treatment; 5) IC plus CCRT as the definitive treatment. The clinical information of all patients was provided in Table 1.

**Table 1:** Clinical characteristics of 341 patients

Characteristics	SUA after two cycles of IC > 327 μmol/L (%)	SUA after two cycles of IC ≤ 327 μmol/L (%)	PR/CR (%)	SD/PD (%)
Total	167 (49.0%)	174 (51.0%)	236 (69.2%)	105 (30.8%)
Gender				
Female	130 (38.1%)	101 (29.6%)	155 (45.5%)	76 (22.3%)
Male	37 (10.9%)	73 (21.4%)	81 (23.7%)	29 (8.5%)
Age (years)				
≤ 50	92 (27.0%)	97 (28.4%)	130 (38.1%)	59 (17.3%)
> 50	75 (22.0%)	77 (22.6%)	106 (31.1%)	46 (13.5%)
Pathological type				
WHO I	0 (0)	2 (0.6%)	0 (0)	2 (0.6%)
WHO II	9 (2.6%)	9 (2.6%)	12 (3.5%)	6 (1.8%)
WHO III	158 (46.3%)	163 (47.8%)	224 (65.7%)	97 (28.4%)
T stage				
T1	24 (7.0%)	17 (5.0%)	28 (8.2%)	13 (3.8%)
T2	32 (9.4%)	38 (11.1%)	52 (15.2%)	18 (5.3%)
T3	65 (19.1%)	72 (21.1%)	99 (29.0%)	38 (11.1%)
T4	46 (13.5%)	47 (13.8%)	57 (16.7%)	36 (10.6%)
N stage				
N0	11 (3.2%)	7 (2.1%)	11 (3.2%)	7 (2.1%)
N1	49 (14.4%)	68 (19.9%)	80 (23.5%)	37 (10.9%)
N2	87 (25.5%)	79 (23.2%)	115 (33.7%)	51 (15.0%)
N3	20 (5.9%)	20 (5.9%)	30 (8.8%)	10 (2.9%)
Chemotherapy cycles				
≤ 3	31 (9.1%)	31 (9.1%)	41 (12.0%)	21 (6.2%)
> 3	136 (39.9%)	143 (41.9%)	195 (57.2%)	84 (24.6%)

*Abbreviations: CR* complete response, *IC* induction chemotherapy, *PD* disease progression, *PR* partial response, *SD* stable disease, *SUA* serum uric acid

The routine pretreatment evaluation covered blood biochemistry, fiber nasopharyngoscopy, MRI of the nasopharynx and cervical region, chest CT scan, abdominal ultrasound, and whole-body bone scanning of the patients. Every patient was re-staged under the eighth edition of the International Union Against Cancer/American Joint Committee on Cancer (UICC/AJCC) guidelines [16].

### Treatment

A total of 341 patients were treated with the definitive IMRT. Details of IMRT in our institution have been described previously [17]. All patients were given IC followed by CCRT. The IC regimen included 2–4 cycles of a platinum-based regimen with 2 or 3 chemotherapeutic drugs every 3 weeks. The recent chemotherapy regimen made up of a single platinum-based drug administered per 3 weeks. Moreover, 112 patients underwent adjuvant chemotherapy, and 43 patients received targeted therapy. Adjuvant chemotherapy and targeted therapy were chosen less often because of poor compliance and high cost to patients with NPC.

### Measurement of the SUA level and tumor response assessment

The SUA level was measured after two cycles of IC using an enzyme kinetics kit (Roche Diagnostics, Germany) by a Modular PP model automated analyzer. Each patient underwent MRI of the nasopharynx as well as cervical region at the pre- and post-induction chemotherapy with two cycles, respectively. Subsequently, the tumor response was assessed by two independent radiologists in a double-blinded manner based on the Response Evaluation Criteria in Solid Tumors criteria 1.1 (RECIST 1.1) [18]. Any tumor response with conflicting results was resolved by consensus. Satisfactory tumor response was determined as complete response (CR) and partial response (PR), whereas unsatisfactory tumor response was classified as stable disease (SD) and disease progression (PD).

### Follow-up and statistical analysis

The follow-up duration was measured from day 1 of the diagnosis of NPC until death or the last follow-up of the patient. The survival endpoints included Overall survival (OS), progression-free survival (PFS), distant metastasis-free survival (DMFS), and locoregional relapse-free survival (LRFS). Beginning from day 1 of treatment, OS was defined as the time to the date of death or patient censoring, whichever occurred first; PFS, to disease failure, death from any cause, or patient censoring, whichever occurred first; DMFS, to distant failure, death from any cause, or patient censoring, whichever occurred first; and LRFS, to local failure or regional failure, death from any cause or patient censoring, whichever occurred first. Patients were evaluated once every 3 months within the first 3 years of follow-up and every 6 months thereafter until death. The median follow-up time was 41 months (range, 3.0–62.0 months).

The SPSS 26.0 software package (SPSS Inc., Chicago, IL, USA) was used for all statistical analyses. Survival analyses were performed by the Kaplan–Meier method, and the log-rank test was employed to make a comparison between the two groups. A Cox proportional hazard model was carried out for the multivariable analyses of the following variables: gender (male versus female), age (≤50 years versus >50 years), pathological type (WHO I-II vs WHO III), T stage (T1-T2 vs T3-T4), N stage (N0–1 vs N2–3), chemotherapy cycle (≤3 versus >3), the SUA level (≤327 μmol/L versus >327 μmol/L), and tumor response to IC (CR/PR versus SD/PD). All statistical tests were 2-sided, and a *P*-value less than 0.05 was confirmed to be statistically significant.

## Results

### Patient characteristics and outcome

Among the 341 patients, the male (*n* = 231)-to-female (*n* = 110) ratio was 2.1:1. The median age of the cohort was 48 (range: 10–78) years. Histologically, 94.1% of the patients had World Health Organization (WHO) type III disease, 5.3% had WHO type II disease, and 0.6% with WHO I type disease. Other clinical characteristics, including T stage, N stage, clinical stage, and chemotherapy cycles, were provided in Table 1.

The median follow-up duration was 41 (range: 3–62) months. By the end of follow-up, 19 (5.6%) patients experienced local or regional recurrence and 49 (14.4%) developed distant metastasis, including 16 cases of pulmonary metastasis, 10 cases of bone metastasis, 14 cases of liver metastasis, 8 cases of multiregional metastasis, and 1 case of metastasis at another site. Finally, 42 (12.3%) patients deceased, including 41 with tumor progression and 1 by car accident.

### Correlation between SUA level after IC and clinical outcome

**Fig. 1:**
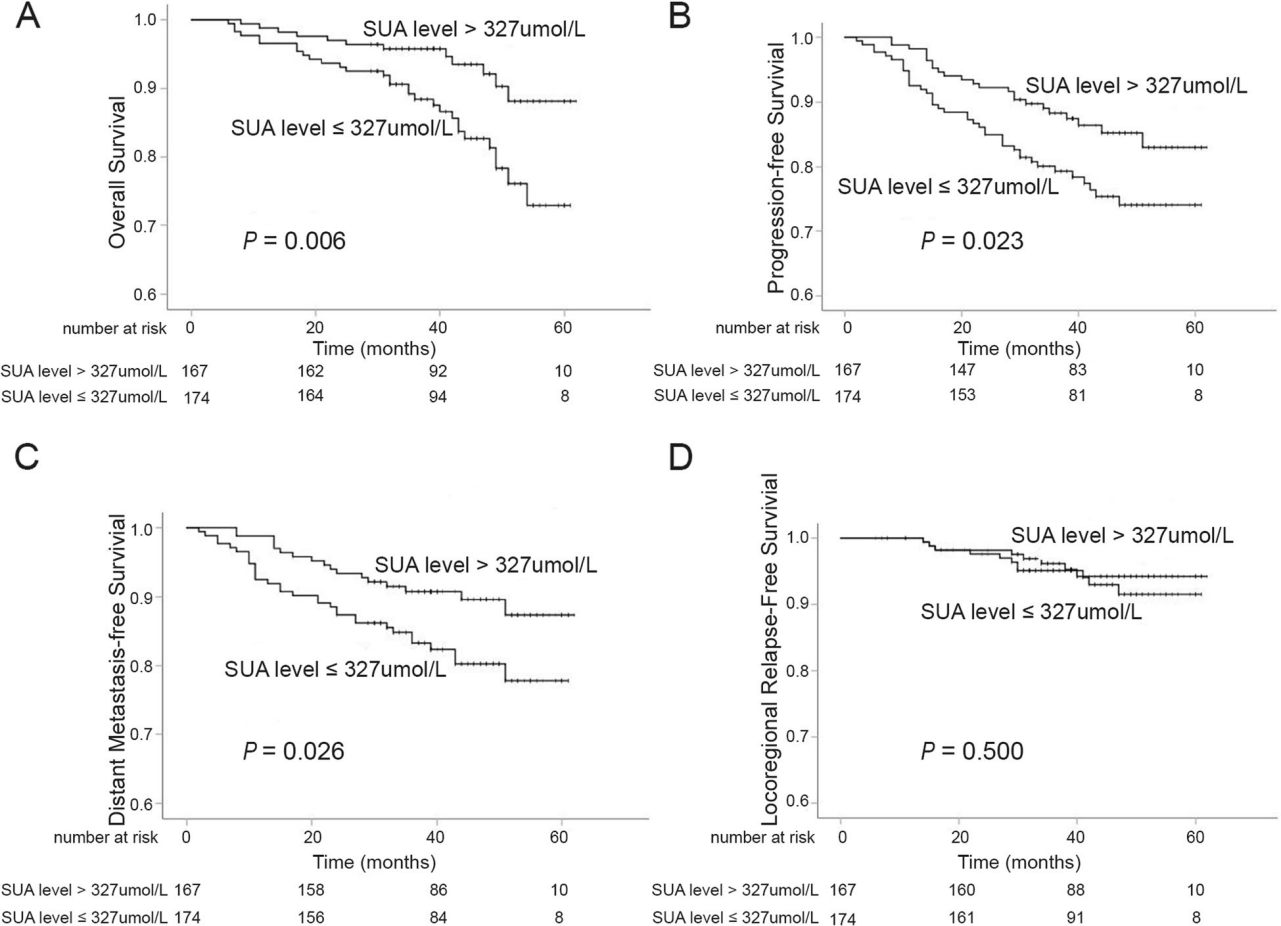
The contrast of OS (**a**), PFS (**b**), DMFS (**c**), and LRFS (**d**) between LANPC patients with high and low SUA level after IC

The median SUA level after two cycles of IC for all patients was 327 (range: 144–585) μmol/L. The 3-year OS, PFS, and DMFS of patients with the SUA level > 327 μmol/L were significantly higher than those with the SUA level ≤ 327 μmol/L after two cycles of IC: 3-year OS, 95.8% (95% confidence interval (CI): 57.4–60.6%) versus 88.4% (95% CI: 52.1–56.5%, *P* = 0.006) (Fig. 1a); 3-year PFS, 88.3% (95% CI: 54.1–58.5%) versus 79.3% (95% CI: 48.4–53.9%, *P* = 0.023) (Fig. 1b); 3-year DMFS, 90.8% (95% CI: 55.7–59.7%) versus 83.2% (95% CI: 50.3–55.5%, *P* = 0.026) (Fig. 1c). However, the 3-year LRFS was not significantly different between the above two groups: 96.2% (95% CI: 58.8–61.4%) versus 95.1% (95% CI: 57.1–59.9%, *P* = 0.500) (Fig. 1d).

### Correlation between tumor response to IC and clinical outcome

**Fig. 2:**
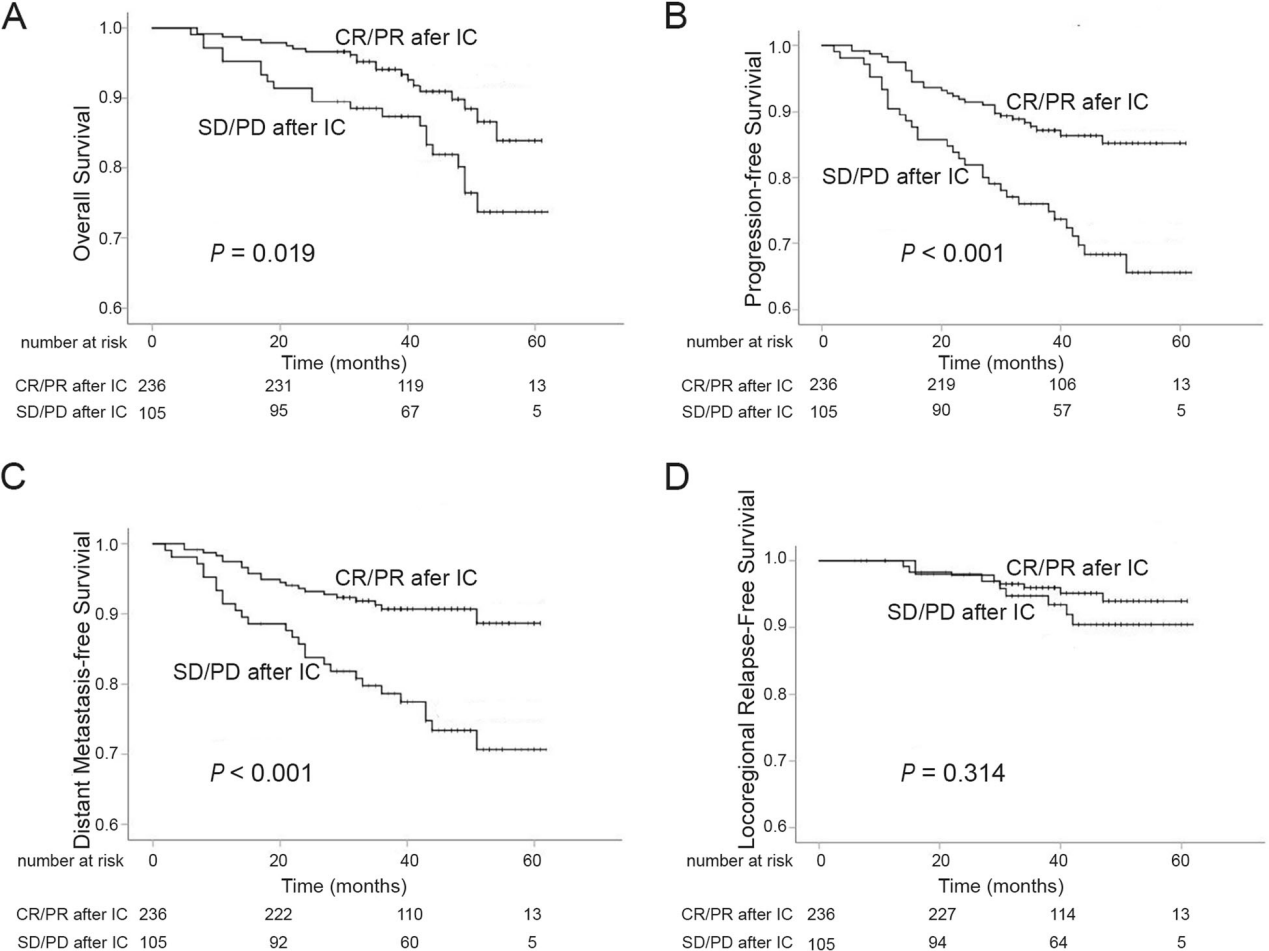
The contrast of OS (**a**), PFS (**b**), DMFS (**c**), and LRFS (**d**) between LANPC patients with a satisfactory and unsatisfactory response to IC

Patients with unsatisfied tumor response had an unfavorable 3-year OS, PFS, and DMFS as a comparison to those with satisfied tumor response: 3-year OS, 94.1% (95% CI: 55.9–58.9%) versus 87.3% (95% CI: 51.3–57.4%, *P* = 0.019) (Fig. 2a); 3-year PFS, 87.1% (95% CI: 53.6–57.3%) versus 76.0% (95% CI: 45.5–53.1%, *P* < 0.001) (Fig. 2b); 3-year DMFS, 90.7% (95% CI: 55.2–58.6%) versus 78.6% (95% CI: 47.6–54.8%, *P* < 0.001) (Fig. 2c). Nevertheless, the 3-year LRFS was not markedly different between the above two groups: 96.0% (95% CI: 58.0–60.2%) versus 94.7% (95% CI: 57.1–61.0%, *P* = 0.314) (Fig. 2d).

### Analysis of the independent predictive factors for LANPC patients after two cycles of IC

To investigate the independent prognostic factors for LANPC patients after two cycles of IC, multivariable analysis was conducted. Consequently, the SUA level after two cycles of IC, tumor response to IC, N stage, and age were found to be independent prognostic factors for OS (*P =* 0.012, *P =* 0.036, *P* = 0.005 and *P* = 0.025, respectively) (Table 2). In addition, tumor response to IC was only observed to be an independent predictive factor for PFS (*P* = 0.001) (Table 2). Furthermore, both the tumor response to IC and N stage were identified as independent prognostic factors for DMFS (*P* = 0.001 and *P* = 0.011, respectively) (Table 2). Notably, the level of SUA was of borderline significance for PFS and DMFS (*P =* 0.055 and *P =* 0.067, respectively) (Table 2).

**Table 2:** Multivariate analyses of independent significance of experimental intervention on clinical outcome

Variables	OS(42/341)		PFS(63/341)		DMFS(49/341)	
	HR (95% CI)	***P***	HR (95% CI)	***P***	HR (95% CI)	***P***
Gender (male vs female)	1.053 (0.546–2.031)	0.877	0.904 (0.518–1.578)	0.723	0.737 (0.384–1.412)	0.357
Age (≤50 vs >50)	2.039 (1.092–3.807)	0.025	1.512 (0.918–2.492)	0.105	1.342 (0.761–2.367)	0.310
Pathological type (WHO I-II vs WHO III)	2.295 (0.361–14.583)	0.379	1.961 (0.531–7.246)	0.313	1.532 (0.420–5.588)	0.518
T stage (T1-T2 vs T3-T4)	1.329 (0.944–1.870)	0.103	1.123 (0.866–1.457)	0.382	1.129 (0.838–1.520)	0.425
N stage (N0–1 vs N2–3)	1.766 (1.186–2.630)	0.005	1.378 (0.990–1.920)	0.058	1.634 (1.117–2.391)	0.011
Chemotherapy cycle (≤3 vs >3)	2.381 (0.910–6.226)	0.077	1.747 (0.830–3.676)	0.142	1.808 (0.767–4.264)	0.176
SUA level (≤327 vs >327)	2.385 (1.208–4.707)	0.012	1.658 (0.989–2.779)	0.055	1.744 (0.964–3.156)	0.066
Tumor response to IC (CR/PR vs SD/PD)	1.915 (1.044–3.648)	0.036	2.381 (1.408–3.816)	0.001	2.764 (1.554–4.851)	0.001

*Abbreviations: CI* confidence interval, *CR* complete response, *DMFS* distant metastasis-free survival, *HR* hazard ratio, *IC* induction chemotherapy, *OS* overall survival, *PD* disease progression, *PFS* progression-free survival, *PR* partial response, *SD* stable disease, *SUA* serum uric acid

### Correlation between combined plasma uric acid level and tumor response and clinical outcome

**Fig. 3:**
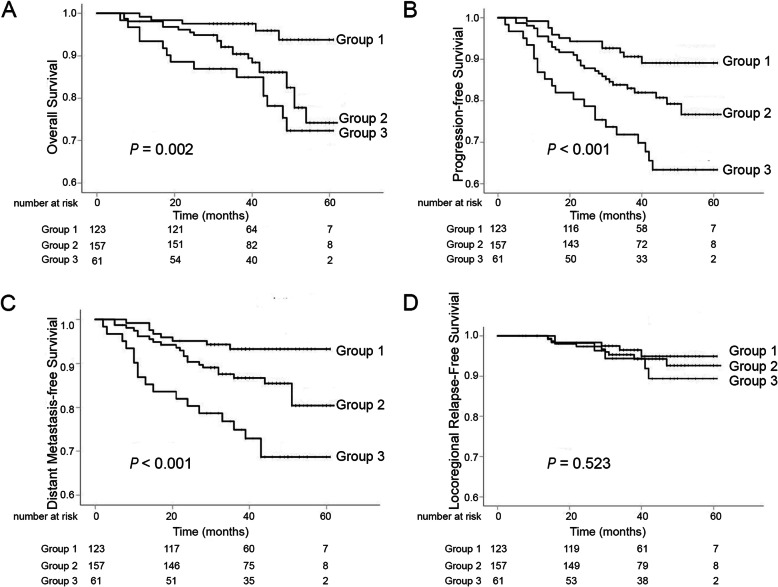
Kaplan–Meier OS (**a**), PFS (**b**), DMFS (**c**), and LRFS (**d**) curves for the combination of SUA and tumor response to IC in LANPC patients. Group1 indicates a high SUA level (> 327 μmol/L) and CR/PR; Group2 indicates a low SUA level (≤327 μmol/L) and CR/PR or a high SUA level (> 327 μmol/L) and SD/PD; Group3 indicates a low SUA level (≤327 μmol/L) and SD/PD

Based on the level of SUA and tumor response, patients were divided into three subgroups: (1) high SUA level (> 327 μmol/L) and CR/PR (*n* = 123); (2) low SUA level (≤327 μmol/L) and CR/PR or a high SUA level (> 327 μmol/L) and SD/PD (*n* = 157); (3) low SUA level (≤327 μmol/L) and SD/PD (*n* = 61). The 3-year OS rates for the three subgroups were 97.6, 90.4, and 84.9%, respectively (*P* = 0.002) (Fig. 3a). The 3-year PFS rates for the three subgroups were 90.6, 83.0, and 71.9%, respectively (*P* < 0.001) (Fig. 3b). The 3-year DMFS rates for the three subgroups were 93.3, 86.7, and 74.9%, respectively (*P* < 0.001) (Fig. 3c). However, the 3-year LRFS rates were similar among the three patient groups: 96.5, 95.3, and 94.4%, respectively (*P* = 0.523) (Fig. 3d).

## Discussion

At present, the addition of IC to the CCRT has been demonstrated as an attractive multidisciplinary approach for the treatment of LANPC [3–5]. Accordingly, few prognostic factors in LANPC patients treated with IC have been investigated. The tumor response to IC was shown to be an independent prognostic factor for LANPC patients underwent IMRT [7, 8]. In addition, the SUA level was confirmed to be elevated because of the radiochemotherapy that led to tumor trauma or lysis [13]. However, the prognostic value of SUA after IC or the SUA levels combined with the tumor response to IC in LANPC patients remains unclear. The current study represents the first report to explore the combined prognostic value of the SUA levels and the tumor response to IC in LANPC patients. In this study, it was shown that a high SUA level after two cycles of IC was involved in significantly improved OS, while PFS and DMFS in LANPC patients exhibited borderline significance. Furthermore, the satisfactory tumor response to IC was correlated with significantly improved OS, PFS, and DMFS in LANPC patients. Moreover, the combination of the SUA level with tumor response to IC represented an optimal predictor of OS, PFS, and DMFS, respectively, in patients with a high SUA level and satisfactory tumor response to IC. These results provided a clinical reference for further guiding the risk stratification and early treatment modification for LANPC.

In this study, both univariate and multivariable analyses revealed that the high SUA level after two cycles of IC (> 327 μmol/L) was a positive prognostic factor for NPC as a comparison to the SUA level ≤ 327 μmol/L with a 2.385-fold increased risk of death. Additionally, borderline significant differences were discovered between the high and low SUA levels with respect to the risk of disease failure and distant failure. However, the SUA level was not correlated with the risk of locoregional failure. Uric acid is considered as an antioxidant, which has the effects of eliminating reactive oxygen free radicals, protecting DNA damage, reducing the cell migration ability, and regulating tumor cell death [11, 19]. Chemotherapy is always associated with increased damage to DNA and reduced tumor burden of the patients, which might finally elevate the SUA levels [20]. In addition, uric acid mediates the cytotoxicity of natural killer and T cells by inducing vital stress-inducing ligands’ expression on cancer cells [21]. Thus, it can be hypothesized that uric acid may exert a preventive effect against the development of cancer. Hence, the SUA levels after chemotherapy reflect the efficacy of chemotherapy in patients with NPC, which leads us to propose that the level of SUA after chemotherapy might be closely related to the prognosis. The level of SUA at the pretreatment and the completion of the definitive IMRT has been reported to be closely tied up with the prognosis in NPC [14, 15]. Nevertheless, additional studies are essential to elucidate the mechanisms associated with high SUA levels after IC and improved survival in LANPC patients treated by IC plus CCRT.

According to the current findings, LANPC patients with SD/PD to IC presented an unfavorable OS, PFS, and DMFS than those with CR/PR to IC. Moreover, the tumor response to IC was identified to be an independent prognostic factor for OS, PFS, and DMFS, respectively. To our knowledge, the prognostic significance of tumor response to chemotherapy in various malignancies has been confirmed [22, 23]. Subsequently, the tumor response to IC was demonstrated to be an independent prognostic factor for LANPC patients with IMRT [7, 8], which was similar to our results. Intriguingly, the tumor response to chemotherapy basically represented the changes in gross tumor volume. Typically, patients with CR/PR to IC implied that the tumor volume shrank dramatically or disappeared completely. In contrast, patients with SD/PD to IC showed that the tumor volume shrank insignificantly or rather increased. Several studies demonstrated the prognostic value of changes in the tumor volume in different types of cancers [24, 25]. Therefore, the prognostic value of tumor response to IC in LANPC patients can be elucidated. Taken together, it can be deduced that the SUA level and tumor response to IC were closely involved in IC, and hence, the combined predictive value of the SUA level and tumor response to IC needs deeper investigation.

To explore the prognostic value of combining SUA levels with tumor response to IC, LANPC patients were divided into three subgroups. The outcomes of recent study showed that the subgroup of patients with a high SUA level and CR/PR exhibited improved OS, PFS, and DMFS; this was defined as the low-risk group. The subgroup of patients with a low SUA level and SD/PD showed poor prognosis and was classified as the high-risk group. These phenomena demonstrated that the combination of SUA levels and tumor response to IC had significant prognostic value in LANPC, which might help in differentiating the risk stratification and improving the prognosis after IC. The high-risk group seemed insensitive to IC, needing more aggressive treatment strategies after IC: (1) an increased dose of RT, (2) the administration of an additional target agent during CCRT, like cetuximab that reported to be a feasible strategy against LANPC [26], (3) the inclusion of adjuvant chemotherapy, or (4) the addition of immunotherapy [27, 28]. For the low-risk group, the radiation dose could be decreased to decrease the side effects of radiotherapy. Furthermore, it is feasible for the treating physicians to decide whether to continue or change the IC regimen based on the SUA level and tumor response after two cycles of IC, which could lower the expenses of the treatment and the complications of chemotherapy.

The current study showed that both the N stage and age were independent prognostic factors for OS by multivariable analyses. In addition, the N stage was also verified to be an independent prognostic factor for DMFS. At present, the N stage is the most crucial risk factor for death and distant metastasis [29]. Furthermore, elderly patients are more likely to develop disease failure and die [30], which is in agreement with the outcome of this study.

Nevertheless, our study has several limitations. First, the tumor response was assessed by the radiologists according to RECIST 1.1 [18]; however, it is hard to evaluate the tumor response in LANPC patients with skull base invasion. In this study, the abnormal MRI signal of the skull base was observed after soft tumor regression, which made it difficult to identify whether the skull base was actually infiltrated. Under such circumstances, the skull base was not included in the measurement of tumor length that may lead to an inaccurate evaluation of the tumor response. Second, the median follow-up duration was only 41 months; thus, it is of great importance to prolong the follow-up time to assess the long-term outcomes of patients with LANPC. Third, other biomedical biomarkers, including plasma EBV-DNA, ALP, and LDH, were not evaluated in our study. Therefore, developing a predictive nomogram model to investigate the array of prognostic factors is imperative.

## Conclusion

In conclusion, the SUA level and tumor response to two cycles of IC have predictive value for LANPC patients receiving IC plus CCRT. However, more aggressive therapeutic strategies are recommended for LANPC patients with a low SUA level and unsatisfactory tumor response to two cycles of IC.

## Data Availability

The data that support the findings of this study are available from Fujian Medical University Cancer Hospital, but restrictions apply to the availability of these data, which were used under license for the current research, and so are not publicly available. Data are, however, available from the corresponding authors upon reasonable request and with permission of Fujian Medical University Cancer Hospital.

## Abbreviations

NPC: Nasopharyngeal carcinoma
CCRT: Concurrent chemoradiotherapy
IC: Induction chemotherapy
LANPC: Locally advanced nasopharyngeal carcinoma
NCCN: National Comprehensive Cancer Network
IMRT: Intensity-modulated radiation therapy
OS: Overall survival
SUA: Serum uric acid
UICC/AJCC: International Union Against Cancer/American Joint Committee on Cancer
RECIST 1.1: Response Evaluation Criteria in Solid Tumors criteria 1.1
CR: Complete response
PR: Partial response
SD: Stable disease
PD: Disease progression
PFS: Progression-free survival
DMFS: Distant metastasis-free survival
LRFS: Locoregional relapse-free survival
CI: Confidence interval
